# Use and Engagement With Low-Intensity Cognitive Behavioral Therapy Techniques Used Within an App to Support Worry Management: Content Analysis of Log Data

**DOI:** 10.2196/47321

**Published:** 2024-01-10

**Authors:** Paul Farrand, Patrick J Raue, Earlise Ward, Dean Repper, Jonathan Baker, Patricia Areán

**Affiliations:** 1 Clinical Education, Development and Research Faculty of Health and Life Sciences University of Exeter Exeter United Kingdom; 2 Department of Psychology Faculty of Health and Life Sciences University of Exeter Exeter United Kingdom; 3 AIMS CENTER Department of Psychiatry and Behavioral Sciences University of Washington Seattle, WA United States; 4 School of Medicine and Public Health Carbone Comprehensive Cancer Center University of Wisconsin-Madison Madison, WI United States; 5 Trent PTS Improving Access to Psychological Therapies Derby United Kingdom; 6 Iona Mind Inc Romford United Kingdom

**Keywords:** cognitive behavioral therapy, low-intensity, mCBT, app, log data, worry management, CBT, management, application, therapy, implementation, treatment, symptoms, anxiety, worry, engagement

## Abstract

**Background:**

Low-intensity cognitive behavioral therapy (LICBT) has been implemented by the Improving Access to Psychological Therapies services across England to manage excessive worry associated with generalized anxiety disorder and support emotional well-being. However, barriers to access limit scalability. A solution has been to incorporate LICBT techniques derived from an evidence-based protocol within the Iona Mind Well-being app for Worry management (IMWW) with support provided through an algorithmically driven conversational agent.

**Objective:**

This study aims to examine engagement with a mobile phone app to support worry management with specific attention directed toward interaction with specific LICBT techniques and examine the potential to reduce symptoms of anxiety.

**Methods:**

Log data were examined with respect to a sample of “engaged” users who had completed at least 1 lesson related to the Worry Time and Problem Solving in-app modules that represented the “minimum dose.” Paired sample 2-tailed *t* tests were undertaken to examine the potential for IMWW to reduce worry and anxiety, with multivariate linear regressions examining the extent to which completion of each of the techniques led to reductions in worry and anxiety.

**Results:**

There was good engagement with the range of specific LICBT techniques included within IMWW. The vast majority of engaged users were able to interact with the cognitive behavioral therapy model and successfully record types of worry. When working through Problem Solving, the conversational agent was successfully used to support the user with lower levels of engagement. Several users engaged with Worry Time outside of the app. Forgetting to use the app was the most common reason for lack of engagement, with features of the app such as completion of routine outcome measures and weekly reflections having lower levels of engagement. Despite difficulties in the collection of end point data, there was a significant reduction in severity for both anxiety (t_53_=5.5; *P*<.001; 95% CI 2.4-5.2) and low mood (t_53_=2.3; *P*=.03; 95% CI 0.2-3.3). A statistically significant linear model was also fitted to the Generalized Anxiety Disorder–7 (*F*_2,51_=6.73; *P*<.001), while the model predicting changes in the Patient Health Questionnaire–8 did not reach significance (*F*_2,51_=2.33; *P*=.11). This indicates that the reduction in these measures was affected by in-app engagement with Worry Time and Problem Solving.

**Conclusions:**

Engaged users were able to successfully interact with the LICBT-specific techniques informed by an evidence-based protocol although there were lower completion rates of routine outcome measures and weekly reflections. Successful interaction with the specific techniques potentially contributes to promising data, indicating that IMWW may be effective in the management of excessive worry. A relationship between dose and improvement justifies the use of log data to inform future developments. However, attention needs to be directed toward enhancing interaction with wider features of the app given that larger improvements were associated with greater engagement.

## Introduction

Excessive worry represents a core characteristic associated with generalized anxiety disorder (GAD) [1], characterized as 6 or more months of chronic worry about several different events and little belief worry can be controlled, and is associated with general somatic symptoms of anxiety [2]. It is highly pervasive in high-income countries, with a lifetime prevalence rate of 7.8% in the United States [3] and a median age of onset between 24 and 50 years, and is more common in women and people who are unemployed [4]. Excessive worry is deleterious to the individual, and if it manifests into GAD, it places a significant burden on society and employers with an average of 6.3 days per month of work absenteeism [5]. Furthermore, treatment is associated with increased service use [6], thereby placing a significant burden on primary care settings within both the United Kingdom [7] and United States [8].

Despite excessive worry impacting on the individual quality of life with progression to GAD representing a major public health problem [9], treatment availability remains limited. The treatment gap for GAD has been reported to be as high as 67% in the United Kingdom and 70% in the United States [10]. In an attempt to address the treatment gap [11], innovations in the delivery of evidence-based psychological therapy have been witnessed, for example, by broadening the workforce delivering cognitive behavioral therapy (CBT) for late-life GAD with no differences in effectiveness demonstrated when delivered by bachelor-level lay providers compared with PhD-level experienced therapists [12]. Further attempts to address the treatment gap have been addressed within the Improving Access to Psychological Therapies (IAPT) program, implementing low-intensity cognitive behavioral therapy (LICBT) self-help for the management of mild to moderate common mental health problems alongside therapist-delivered high-intensity CBT for moderate to severe presentations [13].

While improved access has been facilitated through the IAPT program, predictions indicate that access will only be increased to 25% of the community prevalence of depression and anxiety disorders by 2023-2024 [14]. Furthermore, between 2021 and 2022, only 37% of patients completed a course of therapy [15]. Difficulties in reducing the treatment gap are further dependent on a number of individual-level barriers such as stigma, desire to handle the problem independently, and limited willingness to disclose problems [11].

Greater implementation of digital health technologies such as smartphone apps [16] has potential to address barriers at the level of the individual. Furthermore, with high use of smartphones, for example, 81% of adults within the United States [17], apps offer the promise of delivering mental well-being interventions at scale and derive cost savings with respect to delivery and increased productivity within the workplace [18]. The expansion of apps to manage challenges with well-being has predominantly been based on CBT [19]. CBT is particularly well suited to inform mental well-being apps with emphasis placed on active engagement with specific techniques rather than exclusive reliance on a supportive relationship with a behavioral health coach or therapist [13]. This is especially salient with regard to an approach based on “collaborative empiricism,” whereby after engagement with specific CBT techniques, people are encouraged to explore outcomes for themselves [20]. In doing so, a better understanding of the way their mental health difficulty is affecting them can be derived through an appreciation of the cognitive behavioral model.

Despite CBT providing a compatible evidence-based approach for mental well-being apps with the potential to deliver at scale to close the treatment gap, implementation and uptake remain limited [21]. This is partly dependent on poor engagement with apps relying on factors such as poor usability, techniques inconsistent with user expectations, and poor health literacy [22]. Exploring ways to enhance engagement is of significance given that greater engagement has been reported to predict improvements in mental well-being [23]. In particular, focusing on obstacles and difficulties encountered in completing LICBT techniques is of significance given their effectiveness [24], while not dismissing common factors to establish a good “therapeutic relationship” generating a sense of genuineness, warmth, and collaborative working [25]. Focusing on both common and specific LICBT techniques used within the Iona Mind Well-being app for Worry management (IMWW) is therefore of importance given that the combination of both is crucial for bringing about therapeutic change [26].

This paper reports log data [27] to examine engagement with IMWW based on LICBT to help in the management of excessive worry. It has been proposed that rather than looking at overall engagement regarding areas such as number of sessions engaged with or session duration, it is better to focus attention on specific user interactions [28]. The focus of this paper is therefore directed toward appreciating engagement and interaction with specific LICBT techniques and wider features of IMWW to examine potential enhancements alongside wider usability. Furthermore, the relationship between engagement and outcomes will be explored to inform ongoing development to maximize effectiveness.

## Methods

### Design

Areas representing the focus of log data analysis have been informed by recommendations identified as useful when seeking to address the effectiveness of eHealth technology alongside behavioral and theoretical models [27]. Analysis was only undertaken on data collected regarding “engaged users” and their log data associated with engagement with the LICBT techniques. To be considered an engaged user, the user was required to have completed at least 1 lesson related to the Worry Time and Problem Solving in-app modules within any time period. These criteria represented the “minimum dose” [29] necessary for the user to be considered to have engaged enough to be able to understand the in-app CBT techniques and apply them outside of the app to manage excessive worry. This approach to represent “engaged users” has previously been adopted. For example, with respect to a feasibility trial examining internet-administered LICBT for parents of children treated for cancer [30].

A *χ*^2^ test of homogeneity was undertaken to compare demographic data provided by the engaged users and those who failed to engage with specific LICBT techniques to receive a minimum dose.

### Sample

Over 6 months (July 19, 2022, to February 19, 2023), 956 adults 18 years and older with a GAD-7 score of 6 and above downloaded and opened IMWW and completed the sign-up process. Of these, 803 (84%) adults did not engage sufficiently with the app to be considered an “engaged user,” resulting in 153 (16%) users engaging with the app sufficiently to be considered to have received a minimum dose (Table 1). Approximate data regarding the continent the user was accessing the app from were automatically collected by the app from the time zone set on the user’s phone and therefore collected on all 956 adults.

*χ*^2^ tests of homogeneity indicate that differences between engaged users and those who had downloaded the app but failed to receive a minimum dose were not significant at the 5% level across any of the demographic variables. In addition to the collection of demographic data, 41 of 153 (27%) engaged users responded to a question regarding receipt of other therapy, of whom 34 (83%) users indicated that they were not.

**Table 1:** Demographic questionnaire responses completed (N=956).

Variable		Engaged users (n=153)	Not received minimum dose (n=803)
**Gender, n (%)^a^**			
	Women	43 (72)	50 (85)
	Men	14 (23)	9 (15)
	Other	3 (5)	0 (0)
**Age range (years; n=53), n (%)**			
	18-24	18 (34)	16 (30)
	25-34	20 (38)	19 (36)
	35-44	9 (17)	12 (23)
	45-54	2 (4)	6 (11)
	55-64	1 (2)	0 (0)
	≥65	3 (6)	0 (0)
**Continent, n (%)**			
	Americas	78 (51)	369 (46)
	Europe	33 (22)	196 (24)
	Asia	28 (18)	161 (20)
	Africa	5 (3)	22 (3)
	Australasia	4 (3)	32 (4)
	Unknown	5 (3)	23 (3)

^a^A total of 60 engaged users and 59 users who did not receive the minimum dose responded.

### Iona Mind Well-Being App for Worry Management

The IMWW is, in part, informed by the techniques described in the LICBT written self-help intervention for managing excessive worry [31], based on the CBT protocol for the management of GAD [32]. LICBT is recommended for the management of GAD [33] and is one of the most commonly adopted written self-interventions used within the IAPT program [34]. The focus of IMWW is explained during onboarding where the user is required to explicitly acknowledge its purpose as a well-being tool. Users wishing to continue engagement acknowledge that they understand conditions related to use and consent to have their data processed.

### Collection of Demographic Data

Demographic data were not used to inform the delivery or functionality of the app. Therefore, a screen requesting demographic data, or a question regarding receipt of other therapy, was only presented once the user had engaged with IMWW on 2 occasions at least 6 hours apart following enablement, and no other higher priority messages were pending. If higher priority messages were pending, the request to provide demographic data was repeatedly postponed to the following day until the user had supplied all data, completed specific questions, or declined the request to open the screen (Table 1). Due to the optionality and logic surrounding whether demographic data were requested from users who downloaded the app, such data were therefore not requested from all users and were not prioritized over other more useful app functionality. Collecting demographic data was not prioritized given that answering such questions on an app can increase the risk of disengagement [35].

### Supporting Interaction

Interaction is supported by an algorithmically driven chatbot simulating a “conversation” between the conversational agent (CA) and the user. Users interact by entering raw text or selecting a predetermined response. The type of response depends on the type of content being delivered and varies between selecting a button from a list of options or entering free text when a personalized response is requested. User feedback is collected at the level of a user message supplied within the “conversation” through selecting a “thumbs up” or “thumbs down” icon.

Key principles associated with user-centered systems design were adopted to inform the development of IMWW [36]. The app was developed to manage excessive worry and support emotional wellness with an “SOS” button prominently displayed for users finding themselves in significant emotional distress. If selected, signposting information to a comprehensive list of local and international crisis helplines is presented alongside mindfulness practices to assist with mood stabilization. Before engaging, the user is further reminded that IMWW is not intended to deliver treatment but rather is a tool to support well-being and is not designed for anyone who has been diagnosed with a psychiatric disorder. Consequently, it is stressed that the app is not to be used outside of the context of a well-being self-help aid.

### Progress Through IMWW

Engagement begins with users landing on the Today home screen and progressing through 6 educational modules through which they learn about and interact with the LICBT techniques (Multimedia Appendix 1).

Educational modules are chronologically ordered and unlocked as engagement is initiated. The order in which they are unlocked is dependent on user choice, reflecting whether they wish to initially address practical or hypothetical worries. However, the user is able to move forward and backward between these specific factors to address the different types of worries where preferred. After onboarding, the user is given the opportunity to complete the GAD-7 and Patient Health Questionnaire–8 (PHQ-8) weekly during their weekly review, with scores presented on a progress screen. However, following the completion of these measures during onboarding, subsequent completion is voluntary.

### Home Screen

Informed by the CBT protocol for the management of GAD, which can also be used in the context of improving emotional well-being by supporting the management of worries [37], the Today (Home) screen supports the user to record their worries. This screen also presents a timer that counts down to the user’s scheduled worry time and offers tips to complete Worry Time (Multimedia Appendix 1). Should the user not have completed the lesson, a placeholder is displayed inviting them to learn more about Worry Time. The screen has been designed to make it as easy as possible for users to record worries, plan Worry Time, and access the CBT content. A navigation bar at the bottom of the screen links to the screens related to the LICBT techniques through which users can engage depending on preference.

### CBT Techniques

To promote engagement, the IMWW is informed by collaborative empiricism where the user actively engages with techniques associated with CBT [38]. Collaborative empiricism has been identified as core to the therapeutic relationship [39], supporting “learning by doing” fundamental to CBT [40]. Accordingly, LICBT techniques are presented as skills to be mastered through regular practice. The user is initially introduced to the CBT model followed by supporting them to record and categorize their worries. The user is then provided with the choice of Problem Solving or Worry Time to address practical or hypothetical worries, respectively.

### CBT Model

An interactive CBT Five Areas model (introduced in October 2022) is presented [41], and the user is encouraged to interact and identify a current situation in the “here and now.” In response to this current situation, the user is also encouraged to interact with boxes reflecting “Thoughts” that go through their head, “Behaviors” engaged in, “Physical Feelings,” and to recognize “Emotions” (Multimedia Appendix 1). Additionally, the model helps them appreciate ways in which the specific LICBT techniques presented to address practical and hypothetical worries may be helpful. The model serves as psychoeducation, enabling the user to recognize the interaction between each of these areas, understand the nature of their worry, and appreciate factors that maintain their worry behavior and the impact of physical symptoms associated with anxiety.

### Worry Diary

The primary function of the Worry Diary is to enable the user to actively add new worries as they arise throughout the day and as a record of worries for subsequent review (Multimedia Appendix 1). Prompts and predefined categories are used to enable the user to differentiate between practical worries that have a solution and hypothetical worries that do not.

### Problem Solving

Where practical worries are logged, the user is guided to list potential solutions, consider strengths and weaknesses for each solution, and select the most appropriate one. A time to try the solution out is then optionally scheduled by the user with a reminder given to complete it at the chosen time. After the chosen time has passed or 30 hours elapsed, on opening the app, the CA will ask the user to review how their solution went. Prompts ask the user if the problem was resolved and if not request further information regarding the challenges encountered. Advice is offered where problems have been encountered, putting the solution into action (eg, to break the problem down or work through and apply another solution).

### Worry Time

Unless explicitly overridden by the user, all worries identified as hypothetical are displayed only during Worry Time at a time determined by the user and are blurred out at all other times. Users are reminded that their worry time is starting with a push notification to their phone. If the user opens the app during Worry Time, they are prompted to work through the time they have set aside to worry with the CA. If they choose to do so, the CA will list out all user worries and request that these be worried about for the specified period of time. Subsequently, the CA will review each worry with the user, asking them if it still remains an issue or if it now better represents a practical worry. Worry Time represents a form of cognitive exposure with users exposed to hypothetical worries written down during the day. This is proposed to overcome avoidance behavior and reduce intolerance of uncertainty when it is recognized that there are no solutions to the worry [37].

### Maintaining Engagement

A chatbot informed by theoretically driven techniques is adopted to help establish a “therapeutic approach” to maintain and promote user engagement embedded within IMWW [38]. Such techniques help to establish an approach based on collaborative empiricism [39], whereby the user is encouraged to explore outcomes arising from engagement for themselves.

### Conversational Agent

Support is omnipresent throughout the engagement and comes in the form of an algorithmically driven chatbot stimulating engagement between IMWW, the CA, and users. This helps them overcome difficulties encountered with the specific LICBT techniques and uses common factors to maintain engagement. Upon recognition that difficulties are experienced with any of the specific techniques, the CA is deployed to enable users to work through the specific techniques. If the user reports difficulties in trying out a solution they have planned, the CA will ask questions to determine the nature of the difficulty encountered and direct them to the appropriate parts of the app. For example, the user would be directed back to Problem Solving should they need to break the problem down, or Worry Diary if the worry appears to be hypothetical rather than practical (Table 1).

On other occasions, the CA provides the user with helpful tips and advice or the opportunity to ask FAQs to navigate difficulties experienced. For example, 2 days after learning about Worry Time, the CA will check back in with the user and ask how the exercise has been going. Depending on user response, advice will be given. For example, if the user forgets to engage with Worry Time, they are reminded to turn on their notifications and set an alarm on their phone to serve as a prompt. Consistent with the delivery of CBT, during engagement with the LICBT techniques, the CA brings the user back to the CBT model to reinforce their understanding of the intervention and maintain motivation for continued engagement.

### Common Factors

The CA uses nontherapeutic common factor skills in the form of “therapeutic empathy” to instill a sense of hopefulness and encouragement to maximize engagement with the specific factors linked to symptom reduction [42]. Statements include those demonstrating an empathic stance highlighting a desire to help alongside empathic attunement where statements demonstrate an appreciation of the user’s emotional experience [42]. When recognizing that the user is experiencing difficulties in engaging or is not improving, the CA uses empathy to maintain engagement.

### Behavior Change Techniques

Within the module on recording worries, Behavioral Contracting [43] encourages the user to sign an agreement to consistently engage with IMWW throughout the 6-week program with a separate Goal Setting lesson guiding the user to set approach, rather than avoidance, goals [44]. Behavior “Push” notifications serve to prompt or maintain behavior change while engaging with the app. Furthermore, constructs derived from self-determination theory [45] promote autonomy and intrinsic motivation that serve to facilitate collaborative empiricism [37].

### Monitoring Progress

Throughout engagement, a progress screen presents a summary of the user’s app use and engagement with in-app lessons, previously entered goals, and scores regarding symptom severity associated with anxiety (GAD-7) [46] and low mood (PHQ-8) [47]; it also presents links to the settings page, which houses operational features such as typing speed (Multimedia Appendix 1). Given a potential association between providing feedback and improved outcomes, all data collected are repeatedly presented to the user throughout engagement [48].

### Weekly Reflection

Consistent with face-to-face CBT [39], on a weekly basis, the CA prompts the user to reflect on their engagement with IMWW and the features found most helpful (Table 1). Using reflective learning within the app facilitates learning, with the CA encouraging engagement to promote self-discovery [49]. Furthermore, during the weekly reflection, the CA requests information on the LICBT techniques engaged with and highlights those found most helpful. In the event an identified technique was not engaged with, the CA also requested information as to the main reasons from a range of options provided.

### Data Collection and Analysis

#### Log Data

Consistent with the aims of the study, analysis was undertaken on log data collected from engaged users to reflect their engagement with the specific LICBT techniques. Log data were collected by IMWW automatically logging the actions of each engaged user and requests to complete surveys throughout the use of the app. From these data, summary statistics for use in this paper were extracted. Progress of engaged users through IMWW was monitored and informed by data regarding the number of sessions completed, session duration, weekly reflections, and completion of LICBT techniques alongside summary statistics recorded. Engagement with IMWW was explored with respect to the number of users who reached the CBT model and interacted with it, text entered into each area, completion of the LICBT techniques, and general input and behavior during the engagement. Specific worry management techniques were examined with respect to the number of worries entered and the proportion classified as practical or hypothetical problems. With respect to Problem Solving, data analysis included the number of users who completed the lesson, the number of times the in-app tool was used to solve a practical problem, the number of practical problems entered, the number of users prompted to follow up on their problem-solving with the CA, and the number who engaged with it. Furthermore, analysis was undertaken on the number of engaged users who sought to manage hypothetical worries by learning about Worry Time, set a time for Worry Time, and started an in-app session alongside the number of hypothetical worries entered being recorded.

User responses from the Weekly Reflection conversation within IMWW were also collected and analyzed to gauge general engagement with the specific techniques. As a proxy for behavior change approaches adopted to maintain engagement within the app, the number of users who were delivered at least 1 push notification and the number of those who interacted were also examined. For engaged users completing more than 1 GAD-7 or PHQ-8 at assessment, the log of assessments and the number of times IMWW was used for more than 10 seconds, which is defined as a “session,” were analyzed.

#### Potential Effectiveness

To examine the potential effectiveness of IMWW for engaged users, separate paired samples 2-tailed *t* tests were undertaken to examine the difference between outcome data collected regarding the severity of anxiety (GAD-7) and low mood (PHQ-8). This analysis was only undertaken for the 54 of 153 (35%) engaged users who completed the outcome measures during onboarding and at the end of the engagement.

#### Impact of Engagement on Potential Effectiveness

A multivariate linear regression model was used to investigate the impact that engagement with IMWW had on improvement in anxiety and low mood. In particular, the extent to which specific features were used to complete therapeutic exercises impacted on scores over time. Engagement with, and completion of, Worry Time and Problem Solving was expected to lead to improvements in the symptoms of anxiety, and hence a model to analyze this was specified. Because there are multiple discrete interventions being applied within IMWW and the dependent variable is not univariate, the multiple regression *y*=*β*_0_+*β*_1_*x*_1_+*β*_2_*x*_2_+*ɛ* was adopted. Within this model, *y* is the change in GAD-7 or PHQ-8 from the initial score at onboarding to the final input during progress review, *x*_1_ is the binary variable indicating whether the user completed Problem Solving and resolved their problem, *x*_2_ is the binary variable indicating whether the user completed at least 1 instance of Worry Time in-app, and *ɛ* is the stochastic error term. Additional controls were added to the model to examine the extent to which the number of in-app sessions completed, and the number of worries, problems, and solutions recorded predicted improvement in GAD-7 and PHQ-8. All models met OLS model assumptions associated with multicollinearity, heteroskedasticity, and normality of residuals.

### Ethical Considerations

Users were only able to download IMWW after agreeing to Iona Mind’s Terms of Service and Privacy policy, which required them to acknowledge that they understand conditions related to use and consent to have their anonymized data processed. Being based on anonymous, routinely collected log data from a nonclinical population, research ethics was not required for this study.

## Results

### General Engagement

Analysis of log data collected from the 153 engaged users indicated engagement with 1108 sessions (mean 7.2, SD 7.7) with an average session length of 6.2 (SD 6.2) minutes. The number of sessions and session length varied significantly across users with a median session length of 4.5 minutes and 6 being the median number of sessions (Multimedia Appendix 2).

### Engagement With LICBT Techniques

Since inclusion (October 2022), 36 users started filling out the CBT model to reflect their current difficulties with anxiety, and of these, 31 (86%) users completed all areas in an average of 2.4 minutes (SD 1.7; median 1.7 minutes). The lesson on the Worry Time technique was successfully completed by almost all users (147/153, 96%). However, of these users, only 50 (33%) were observed to have performed Worry Time at their chosen time using the in-app tools.

Problem Solving was engaged with by 114 of 153 (74.5%) users; however, only 89 of 153 (58.2%) users actually completed the lesson. This indicates that 25 of 114 (21.9%) users engaged with the in-app tools to problem-solve one of their practical worries without completing the lesson. This behavior is permitted within the IMWW user experience because the user is able to choose the specific features of the app they wish to engage with. The majority of app features start in an unlocked state to encourage exploration and self-discovery. Only 42 of 153 (27%) users completed a follow-up conversation to review their solutions and progress using the Problem Solving protocol on their worries. During the interaction, engaged users recorded a total of 720 worries (mean 4.7, SD 6.1), and a median of 3 worries were recorded for each user. Of the worries recorded, 399 (55%) were categorized by the user as practical, 306 (43%) as hypothetical, and only 15 (2%) worries were not categorized. With respect to practical worries, 244 (61%) worries were problem-solved using the in-app tools with at least 1 possible solution added.

### Weekly Reflection

In response to the CA asking the user to reflect on their experience of engaging with IMWW, in-app Weekly Reflections were completed by 58 of 153 (38%) users who recorded 206 responses (mean 3.6) identifying LICBT techniques engaged with, alongside 48 responses identifying the technique found most helpful (Table 2).

During the Weekly Reflection, 27 of 58 (47%) individual users reflected on engaging with Worry Time, of whom 19 (70%) were observed to have used the in-app tooling to complete it at their chosen time. A total of 8 of 58 (14%) users therefore engaged with Worry Time without using the in-app tools. In addition to asking which features of IMWW the user had engaged with, the CA also asked which feature they found most helpful. The users were asked this question during the weekly review, and for each weekly review, they could give at most 1 response.

**Table 2:** Weekly reflection techniques engaged with and found most helpful.

Technique	Engaged with^a^ (n=206), n (%)	Most helpful (n=48), n (%)
Journaling worries	50 (24)	16 (33)
Worry time	46 (22)	11 (23)
Problem-solving	35 (17)	10 (21)
Avoiding worry behaviors	28 (14)	4 (8)
Watching out for different worry types (Worry categorization)	17 (8)	4 (8)
CBT^b^ model^c^	8 (4)	1 (2)

^a^Users can respond multiple times.

^b^CBT: cognitive behavioral therapy.

^c^Introduced in October 2022.

### Maintaining Engagement

To maintain engagement with IMWW, 142 of 153 (93%) users were sent at least 1 push notification with 113 of 153 (74%) users responding. An average of 84 (SD 75) push notifications were sent to each engaged user throughout their engagement, although the quantity of push notifications per user varied substantially with use pattern and duration.

### Potential Effectiveness

**Figure 1:**
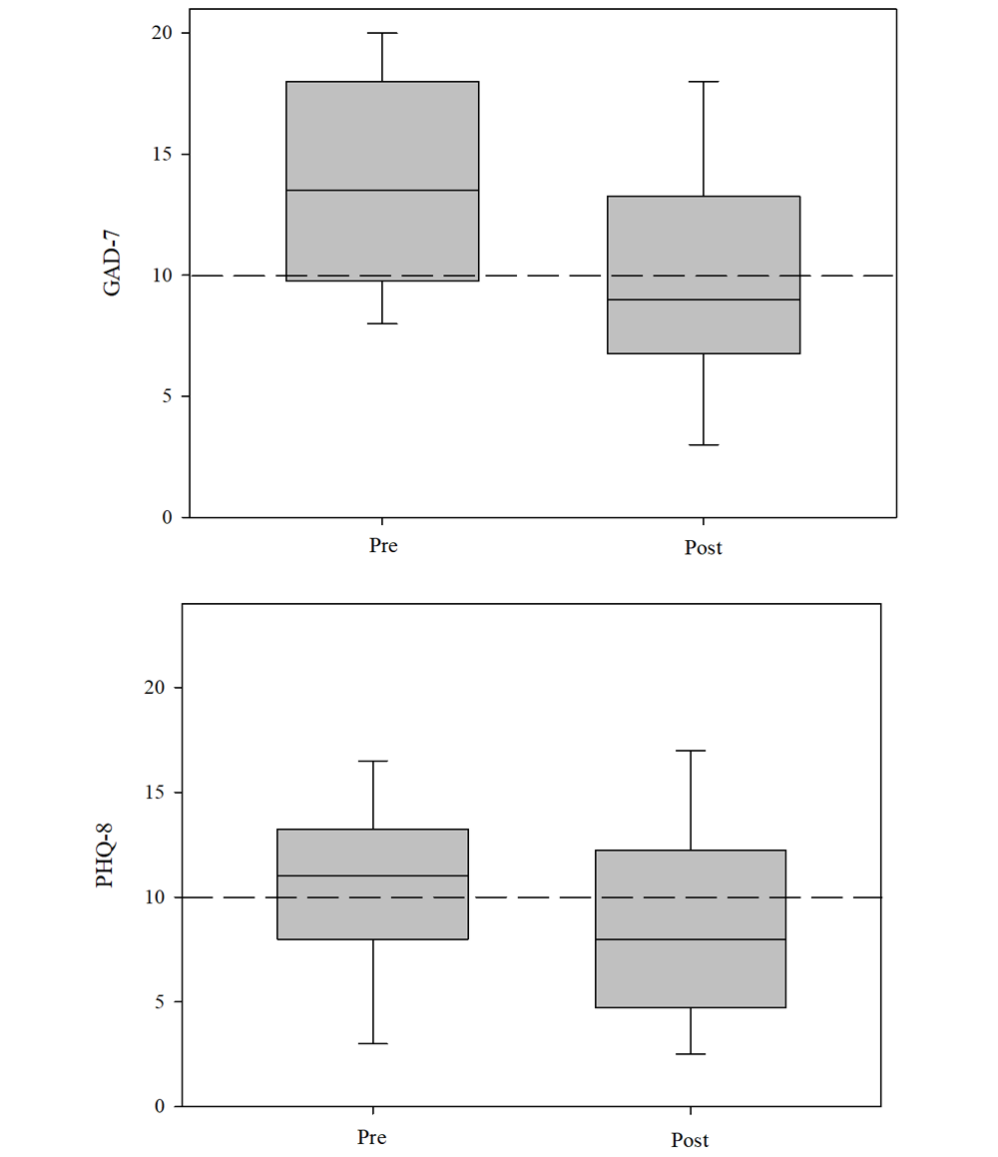
Pre-post mean differences (95% CI) for GAD-7 and PHQ-8. GAD-7: Generalized Anxiety Disorder–7; PHQ-8: Patient Health Questionnaire–8.

Separate paired sample 2-tailed *t* tests were conducted to examine the difference between the GAD-7 and PHQ-8 scores for 54 of 153 (35%) engaged users who completed the measures during onboarding and the final score provided. There was a significant reduction in both anxiety (t_53_=5.5; *P*<.001; 95% CI 2.4-5.2) and low mood (t_53_=2.3; *P*=.03; 95% CI 0.2-3.3), with severity dropping from moderate to mild in both instances (Figure 1).

Examination of individual-level data indicates that the vast majority of users (43/53, 81%) experienced a reduction in anxiety between baseline and final observation with the score of 2 (4%) users remaining unchanged. The majority of users (35/53, 66%) also saw a reduction in PHQ-8 with no difference arising for 4 (8%) users. Deterioration in GAD-7 was experienced by 9 (17%) users and rose to 15 (28%) users for low mood.

### Impact of Engagement on Potential Effectiveness

The multivariate linear regression predicting changes in GAD-7 based on engagement reached statistical significance (*F*_2,51_=6.73; *P*=.03), but the model predicting changes in the PHQ-8 did not (*F*_2,51_=2.33; *P*=.11). Two-sided 2-tailed *t* tests were performed on the slope estimates in the models. The model predicting changes in GAD-7 estimates that the marginal effect of a user completing in-app Worry Time (*β*_2_) is a –3.3 change in GAD-7 and is significant (*P*=.02). The constant *β*_0_ and the marginal effect of the user successfully completing Problem Solving *β*_1_ had respective values of –1.6 and –3.0. However, they failed to reach significance at α=.05 (*P*_0_=.07, *P*_1_=.08). The model had an *R*^2^ of 0.21. Furthermore, an improvement in the GAD-7 and PHQ-8 was not predicted by the number of in-app sessions completed (*P*=.09) or the number of worries (*P*=.36), problems (*P*=.27), and solutions (*P*=.16) recorded.

## Discussion

### Principal Findings

While engaged users represented a minority of those who downloaded IMWW, a large number of these interacted with the LICBT techniques associated with the CBT protocol to manage excessive worry and support emotional well-being [31]. The vast majority of those who engaged completed all areas presented with the CBT model and were able to successfully record worries and categorize them as practical or hypothetical. The CA was commonly used to help engaged users overcome difficulties when engaging with practical worries. Worry Time was engaged with to a much lesser extent within the app; however, several users reported engaging with it outside of the app. Forgetting to engage with the LICBT techniques was identified as the most common reason for lack of engagement, while experiencing them as too difficult to comprehend was only reported by a small minority of engaged users. The change in the user GAD-7 score was predominantly explained by engagement with the LICBT techniques as opposed to the number of times they used IMWW.

Poor engagement with an app following download is not uncommon, with only 14% of people often using it the following day [50] and even lower rates typically associated with mental health apps [22]. Despite using common factors and behavior change techniques, however, only a minority of users who engaged with IMWW had enough engagement with the LICBT techniques to be considered engaged users. This is of some concern given that users failing to engage to a point where they have received an adequate dose to bring about recovery may serve as a barrier to seeking further support.

While engagement following download was poor, log data identified that engaged users had moderate to good levels of interaction and fidelity [51], with the CBT model alongside recording and categorizing worries. Fidelity and interaction with Problem Solving were also good, potentially arising from support provided through the CA. When engaging with Problem Solving, the CA was commonly used to support users to overcome difficulties in engaging with the LICBT techniques and to encourage continued engagement. There was less within-app engagement with Worry Time; however, some users reported engaging with it outside of the app. Engagement with the LICBT techniques included within IMWW may therefore have been greater than log data alone suggest. This supports the additional benefits of exploring out-of-app engagement with specific techniques to get a full appreciation of interaction [28]. Exploring ways to promote out-of-app engagement is of benefit given that engagement with techniques in face-to-face CBT between support sessions as “homework” is identified as important to improve clinical outcomes related to anxiety [52].

Although there were moderate levels of interaction with LICBT techniques used within IMWW, exploring additional ways to enhance interaction across all techniques and promote prolonged engagement would be highly beneficial. Enhancing engagement through approaches such as involving personalized support, guidance, and feedback regarding engagement has also been associated with improved effectiveness for mental well-being digital tools [48]. Furthermore, recommendations to enhance out-of-app homework compliance to deliver better outcomes have also been proposed [53]. These include ensuring that app content is congruent to the therapeutic approach adopted, learning is consolidated through engagement, and emphasis is placed on completion. Additionally, recommendations include ensuring that the app is tailored to specific populations and building connections with others has been identified as supporting engagement with homework [53]. Within IMWW the CA was used to enhance engagement through the use of common factor skills to encourage and motivate the user. However, greater focus needs to be directed toward maximizing the ability of the CA to enhance engagement within and outside of the app.

Maximizing engagement may be achieved by implementing mental well-being apps for use adjuvant to health professional support and integrated into clinical settings [54]. Benefits associated with providing support are recognized by the National Institute of Health and Care Excellence recommendations for supported LICBT for anxiety and depression [14]. This has resulted in Psychological Practitioner support for LICBT adopted by the IAPT program implemented across England [14]. Support enables the patient to engage with the interventions by using personalized common factor skills, monitor progress, and provide encouragement during weekly support sessions. However, it does not include a therapeutic role in the delivery of LICBT techniques within the clinical sessions [13].

However, nonprofessional forms of support have also been demonstrated to enhance engagement and improve outcomes with LICBT. For example, group support within community settings is provided by trained volunteers with varying backgrounds [55]. Furthermore, forms of support through technology such as web-based communities providing constructive peer support [55] and discussion forums [56] have been identified to enhance engagement with digital tools [54]. Potentially, therefore, using IMWW adjuvant to some form of minimal-contact support provided by a practitioner, volunteers within community organizations, or mediated through technology offers promise to result in enhanced effectiveness at reduced delivery costs.

With respect to outcomes, the average level of anxiety and low mood improved among users who engaged with IMWW to a degree they would be considered to have received a minimum dose of the LICBT techniques [29]. That anxiety and low mood are identified to share mechanisms has led to recommendations to combine techniques within a single app to reduce the commitment needed by users to maximize engagement [19]. However, when exploring recovery at the level of the individual user, the low mood of several more users deteriorated compared with anxiety. However, it would remain possible to develop a single app that included protocol-informed LICBT techniques to target low mood or anxiety once the main emotional difficulty being experienced was determined.

### Strengths and Limitations

Providing a clear description of the LICBT techniques contained within IMWW informed by a theoretical basis represents a real strength of the paper. This has enabled the analysis of log data to be interpreted with respect to interactions with the techniques. Clearer conclusions regarding the relationship between engagement and outcomes regarding the management of symptoms associated with anxiety were therefore able to be reached. This facilitates specific targeting of future development work on IMWW to ensure greater levels of engagement to derive improved outcomes.

There was a large difference between the number of people who downloaded IMWW and those who interacted with at least 1 lesson related to Worry Time and Problem Solving for them to be considered engaged users. While it is known that the background demographics of these 2 groups did not significantly differ, it is unclear as to why a large number of those who downloaded IMWW never went on to engage with one of these specific LICBT techniques. Unfortunately, reasons behind failing to engage with IMWW were not requested, and therefore the extent to which poor usability may have been a relevant factor is unknown. As the use of digital health technologies continues to increase [16], understanding the usability of apps is of increasing interest [57]. Future research exploring log data could therefore consider using a measure of usability, such as the mHealth Usability Questionnaire [58], alongside the collection of log data to gain a better understanding of the way in which an app is used alongside potential barriers to usability.

Finally, while data regarding outcomes can be considered promising with respect to IMWW as a tool to support worry management, this study does not enable definitive conclusions regarding effectiveness to be reached. As a consequence of the lack of clear end points when using log data, users can stop using the app at any time without completing outcome measures. This makes it difficult to reach conclusions regarding effectiveness. The use of multivariate regression with terms to represent proxy use of techniques was adopted to compensate for this. However, this cannot be considered to represent a substitute for the collection of clear and reliably collected end point data within a trial design comparing IMWW with an appropriate control [59]. Furthermore, reaching conclusions regarding effectiveness is further confounded given that only a minority of engaged users responded to a question regarding the current receipt of treatment.

### Conclusions

While a large number of people downloaded IMWW, only a minority engaged with the app to be considered engaged users. Of these users, however, analysis of log data identified good interaction with the LICBT techniques associated with an evidence-based protocol to support worry management [31]. Although there were good levels of interaction, exploring additional ways to promote interaction with the LICBT techniques and other features of the app to result in prolonged engagement remains beneficial. This could involve adopting a “user-centric” design process whereby potential users are directly involved in ongoing development [22]. Considering log data as part of a user-centric design process may enhance engagement to a point where more users receive an appropriate “dose” to bring about improvement [29]. Log data can therefore be used to inform ongoing development to maximize engagement and protocol fidelity [51]. This is significant given the relationship between engagement and effectiveness. While effectiveness data associated with IMWW can only be seen as promising, capturing log data will serve to enhance ongoing intervention development. A high-quality randomized controlled trial would then enable definitive conclusions regarding effectiveness to be reached [54]. This would help address concerns that the current level of evidence derived from poor-quality trials does not enable recommendations regarding apps to enhance mental well-being to be reached [60].

## Appendix

Multimedia Appendix 1 Progress through the Iona Mind Well-being app for Worry management.

Multimedia Appendix 2 Number of sessions of varying length.

## Abbreviations

CA: conversational agent
CBT: cognitive behavioral therapy
GAD-7: Generalized Anxiety Disorder–7
IAPT: improving access to psychological therapies
IMWW: Iona Mind Well-being app for Worry management
LICBT: low-intensity cognitive behavioral therapy
PHQ-8: Patient Health Questionnaire–8
