# Correction: Use and Engagement With Low-Intensity Cognitive Behavioral Therapy Techniques Used Within an App to Support Worry Management: Content Analysis of Log Data

**DOI:** 10.2196/76573

**Published:** 2025-05-13

**Authors:** Paul Farrand, Patrick J Raue, Earlise Ward, Dean Repper, Jonathan Baker, Patricia Areán

**Affiliations:** 1 Clinical Education, Development and Research Faculty of Health and Life Sciences University of Exeter Exeter United Kingdom; 2 Department of Psychology Faculty of Health and Life Sciences University of Exeter Exeter United Kingdom; 3 AIMS CENTER Department of Psychiatry and Behavioral Sciences University of Washington Seattle, WA United States; 4 School of Medicine and Public Health Carbone Comprehensive Cancer Center University of Wisconsin-Madison Madison, WI United States; 5 Trent PTS Improving Access to Psychological Therapies Derby United Kingdom; 6 Iona Mind Inc Romford United Kingdom

In “Use and Engagement With Low-Intensity Cognitive Behavioral Therapy Techniques Used Within an App to Support Worry Management: Content Analysis of Log Data” (JMIR Ment Health 2024;12:e47321), the authors made the following changes.

The authorship was previously published as:

Paul Farrand^1,2*^, PhD; Patrick J Raue^3*^, PhD; Earlise Ward^4*^, PhD; Dean Repper^5*^, MSc; Patricia Areán^3*^, PhD

The following author, equal contribution tag, ORCID, and associated affiliation have been added in the fifth position of the authorship:

Jonathon Baker^6*^, MA (ORCID 0009 0000 9923 5042)

Iona Mind Inc, Romford, United Kingdom

The Authors' Contributions was revised from:

PF, PA, and PJR conceptualized and designed the project with EW and DR providing theoretical input informing the background of the paper. PF wrote the initial draft of the paper with all authors contributing to the development of the paper, interpretation of the analysis, and editing of the final manuscript. PF was not involved in any part of data analysis.

To:

PF, PA, and PJR conceptualized and designed the project with EW and DR providing theoretical input informing the background of the paper and JB extracting and organising data for analysis, drafting the section describing the app and clarifying any uncertainties regarding description of the app in relation to descriptive log-data. PF wrote the initial draft of the paper with all authors contributing to the development of the paper, interpretation of the analysis, and editing of the final manuscript. PF and JB were not involved in any part of data analysis.

Additionally, the Conflicts of Interest section previously appeared as:

PF is currently on a paid sabbatical with Iona Mind from the University of Exeter. All other authors have confirmed they have no conflicts of interest to declare.

And will now be changed to:

JB is a full time employee and PF currently on a paid sabbatical with Iona Mind from the University of Exeter. All other authors have confirmed they have no conflicts of interest to declare.

The correction will appear in the online version of the paper on the JMIR Publications website, together with the publication of this correction notice. Because this was made after submission to PubMed, PubMed Central, and other full-text repositories, the corrected article has also been resubmitted to those repositories.

