# Supplementary figures and images for: Use and Engagement With Low-Intensity Cognitive Behavioral Therapy Techniques Used Within an App to Support Worry Management: Content Analysis of Log Data
Source: JMIR Mhealth Uhealth. 2024 Jan 10;12:e47321. doi: 10.2196/47321 (PMC10809068; doi:10.2196/47321)

Multimedia Appendix 1. Progress through IMWW


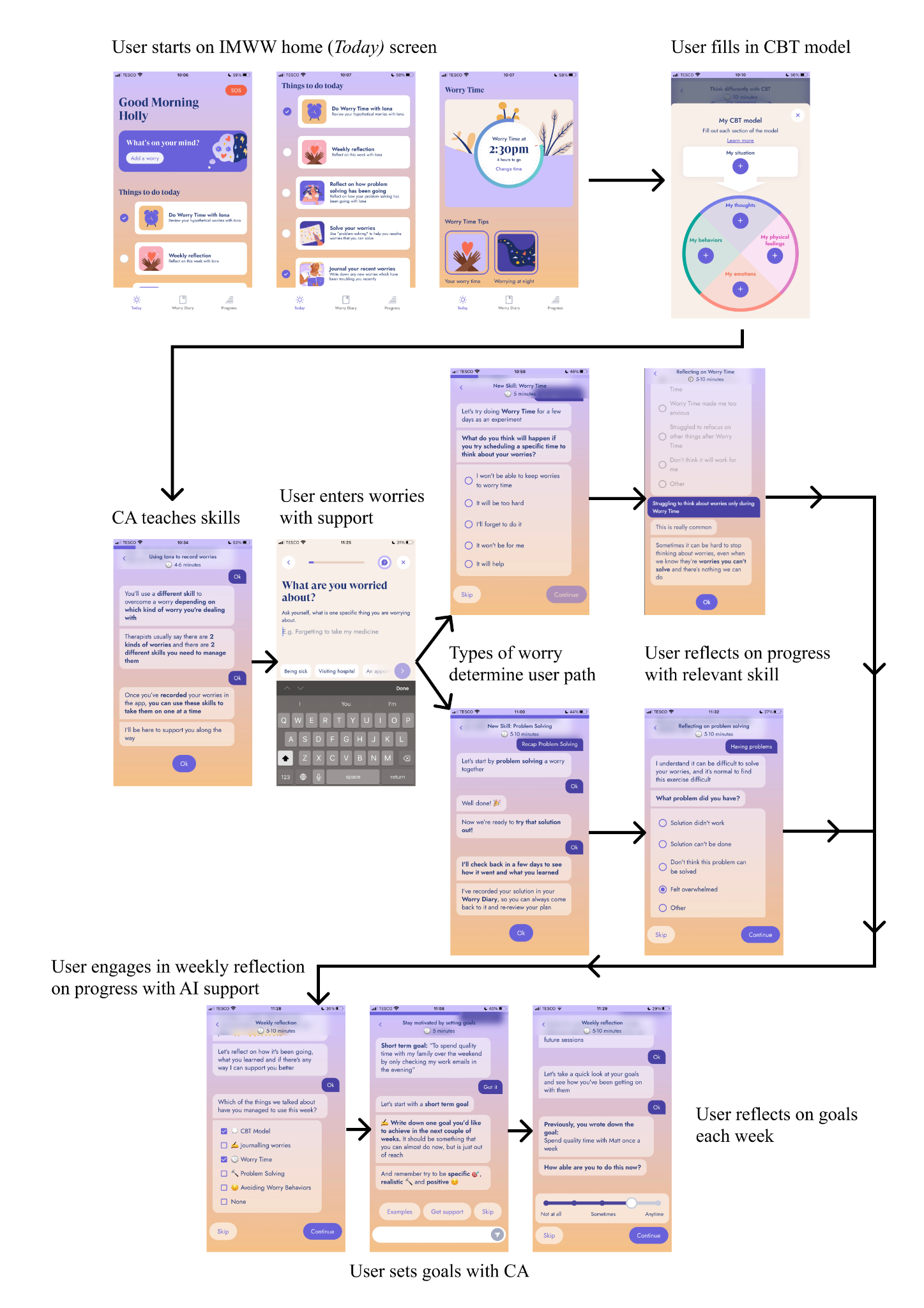

Supplement: Multimedia Appendix 1 [file mhealth_v12i1e47321_app1.docx]

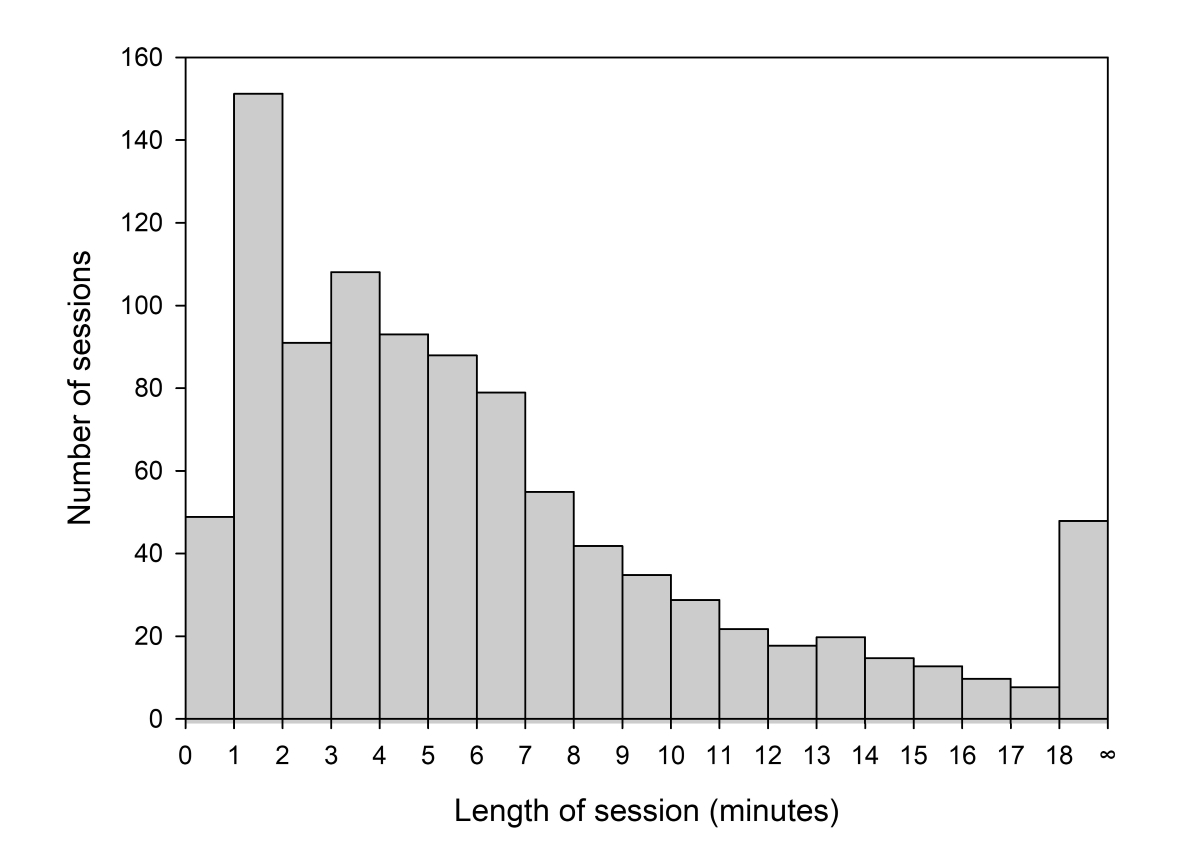

Supplement: Multimedia Appendix 2 [file mhealth_v12i1e47321_app2.png]
